# Intercalation Polymerization Approach for Preparing Graphene/Polymer Composites

**DOI:** 10.3390/polym10010061

**Published:** 2018-01-10

**Authors:** Yifan Guo, Fuxi Peng, Huagao Wang, Fei Huang, Fanbin Meng, David Hui, Zuowan Zhou

**Affiliations:** 1 Key Laboratory of Advanced Technologies of Materials (Ministry of Education), School of Materials Science and Engineering, Southwest Jiaotong University, Chengdu 610031, China; yfguo@my.swjtu.edu.cn (Y.G.); fuxipeng@my.swjtu.edu.cn (F.P.); huagaowang@163.com (H.W.); fhuang0137@163.com (F.H.); 2 Department of Mechanical Engineering, University of New Orleans, New Orleans, LA 70148, USA; dhui@uno.edu

**Keywords:** graphene/polymer composites, intercalation of graphite, exfoliation intercalation polymerization, interaction

## Abstract

The rapid development of society has promoted increasing demand for various polymer materials. A large variety of efforts have been applied in order for graphene strengthened polymer composites to satisfy different requirements. Graphene/polymer composites synthesized by traditional strategies display some striking defects, like weak interfacial interaction and agglomeration of graphene, leading to poor improvement in performance. Furthermore, the creation of pre-prepared graphene while being necessary always involves troublesome processes. Among the various preparation strategies, an appealing approach relies on intercalation and polymerization in the interlayer of graphite and has attracted researchers’ attention due to its reliable, fast and simple synthesis. In this review, we introduce an intercalation polymerization strategy to graphene/polymer composites by the intercalation of molecules/ions into graphite interlayers, as well as subsequent polymerization. The key point for regulating intercalation polymerization is tuning the structure of graphite and intercalants for better interaction. Potential applications of the resulting graphene/polymer composites, including electrical conductivity, electromagnetic absorption, mechanical properties and thermal conductivity, are also reviewed. Furthermore, the shortcomings, challenges and prospects of intercalation polymerization are discussed, which will be helpful to researchers working in related fields.

## 1. Introduction

Graphene, a single atom-thick sheet composed of sp^2^-hybridized carbon, has received considerable attention since its first fabrication through mechanical exfoliation in 2004 [1]. Favored for its unique two-dimensional structure and extraordinary electrical [2,3], thermal [4,5], mechanical properties [6,7,8,9], graphene is widely researched in energy storage and conversion, spintronic devices, photonics and optoelectronics and other kinds of materials. In recent years, the hybridization or composites based on graphene and its derivatives has attracted much interest in physics, chemistry and materials domains. Among this research, the introduction of graphene in polymer significantly increases Young’s modulus [10,11,12], and electrical [13,14,15] or thermal conductivity [16,17,18] of polymer composites, particularly at low volume fractions (<1 wt %). Moreover, some special properties of the polymer composites such as shape memory [19,20,21], chemiluminescence [22] and microwave absorption [23,24,25,26] may emerge, resulting from the interaction between graphene and polymer.

Melting blend and solution mixing are the most economically attractive and scalable methods for prepared graphene/polymer composites [27,28,29,30,31]. However, agglomerate pre-prepared graphene is always hard to disperse in polymer melt or solution because of their high viscosity. Moreover, interfacial interactions between the graphene and polymer matrix are weak, resulting in low enhancement of polymer properties. In situ polymerization after the dispersion of graphene in a monomer is another way to synthesize graphene/polymer composites [32,33]. On the one hand, a particular monomer can be used to disperse graphene in the system [34]. On the other hand, dispersed graphene layers act as the hard template of polymerization, leading to strong intercalation between graphene and polymer [35,36].

Pre-prepared graphene is needed when the aforementioned methods are used to process the graphene/polymer composites. According to recent reports, bottom-up approaches, including chemical vapor deposition (CVD) [37,38] and epitaxial growth [39], are widely used to produce high-quality graphene. Although large crystal domain, specific layer graphene can be synthesized via tuning of carbon source and growth conditions, the high cost and low yield of these methods associated with difficulties in exfoliating graphene from the substrate limit their application in industrial production. Therefore, most of the graphene used in the further processes is produced by exfoliation of natural graphite (NG) or highly ordered pyrolytic graphite, named top-down approaches. Among these approaches, dry exfoliation performed by using mechanical, electrostatic or electromagnetic forces can result in grain boundary-free graphene [1]. However, these approaches are impractical for large-scale applications. The thickness and size of graphene layers can hardly be controlled, and it is thus unsuitable for use in composite preparation. By comparison, sonication-assisted liquid phase exfoliation in reasonable solvents has been considered as one of the most promising routes for the mass production of low-cost and high-quality graphene [40,41,42]. However, the long time required for sonication and the further purification process (always involving ultracentrifugation) may limit the production period when applied in industry-scale production. For the reduction of graphene oxide (GO), the synthesis of graphite oxide (GtO) always involves successive oxidative treatments containing a strong acid and oxidant. Only in recent years have some efforts been made to avoid of using such environmentally damaging substances [43,44]. Moreover, chemical oxidation always introduces an oxygen-containing functional group in the basal plane or edges, acting as active sites for further modification and functional applications such as biosensing, catalytic, electromagnetic waves absorption, supercapacitors etc. [45] On the other hand, oxidation of graphene leads to damage of the basal plane, thus degrading some properties relying on the perfect crystalline structure (typically tensile strength and electrical or thermal conductivity) [44]. But the π–π conjugate can be partially recovered relying on the reduction degree of GO [46]. Furthermore, even if high-quality graphene could be produced on a large scale, the pre-prepared graphene powder or concentrated slurry is always difficult to disperse uniformly whether in a polymer matrix or monomer solution. This usually results in a limited performance improvement in graphene/polymer composites.

In recent years, the exfoliation of graphite intercalated compounds (GIC) has been deemed another interesting approach to realizing the exfoliation of graphite to the graphene layer. GIC is formed by insertion of particular atomic or molecular layers between the layers of graphite. The weak Van der Waals interaction, a distance of 3.35 Å, and an abundant π-electron cloud between graphite layers ensure the intercalation process of alkali metal [47,48], sulphuric [49] and some metal chloride [50,51]. The graphene layer can be then easily exfoliated with the assistant of mechanical or heat treatment [52]. This approach has gained attention due to easily available raw materials, its simple operation and high-quality products. Furthermore, industry-scale production can be expected to be based on this method.

Inspired by the exfoliation of GIC, in situ intercalation polymerization using organic monomers was recently proposed for synthesizing a graphene/polymer composite in a one-step process [53]. Benefiting from an abundant π-electron cloud, different kinds of monomer cation can penetrate into the interlayer of graphite and subsequently polymerize in the gap of the planes. While the intercalants weaken the inter-planar bonding, polymerization then separates the layers from the intergallery, resulting in the formation of graphene/polymer composites. Interest in the reliable, fast and simple synthesis means that intercalation polymerization has gained more attention in the strategies of graphene/polymer composites preparation. Therefore, how to intercalate molecules/ions/clusters into graphite, and how to conduct polymerization in the graphite interlayers, are now research topics. In this review, we discuss the recent progress of the intercalation of graphite, including inorganic-GIC mostly synthesized by two-zone vapor transport and electrochemistry methods; and organic intercalating compounds synthesized by electrochemistry, cation exchange or chemical methods. Furthermore, polymerization conducted in graphite interlayers, which can be divided into monomers initiated by pre-intercalated compounds and polymerization of intercalative monomers (in situ intercalation polymerization), are reviewed here. Some regular results, shortcomings, challenges, and prospects of intercalation methods and interlayer polymerization are also suggested. Potential applications of graphene/polymer composites prepared by intercalation polymerization, including electrical conductivity, electromagnetic absorption, mechanical properties and thermal conductivity, are introduced, which will be helpful to people working in related fields.

## 2. Intercalation of Graphite

### 2.1. Traditional Graphite Intercalated Compounds (GIC)

The capture of organic monomers in the interlayer of graphite is the prerequisite for intercalation polymerization and the consequent exfoliation of graphene. Therefore, the intercalation of molecules, ions or clusters is one of the key issues in the process. In fact, the intercalation of graphite has been researched for more than one hundred years since the first synthesis of GIC reported by Schaffäutl (1841). Owing to the layered structure, natural graphite provides shelter for guest molecules with subnanometer interlayer distance. While graphite can act as an electron donor or acceptor based on the reaction conditions [54], hundreds of kinds of atomic and molecular layers with various physical/chemical characteristics, have been intercalated into the interlayer space of graphite host material to form GIC [55].

**Figure 1:**
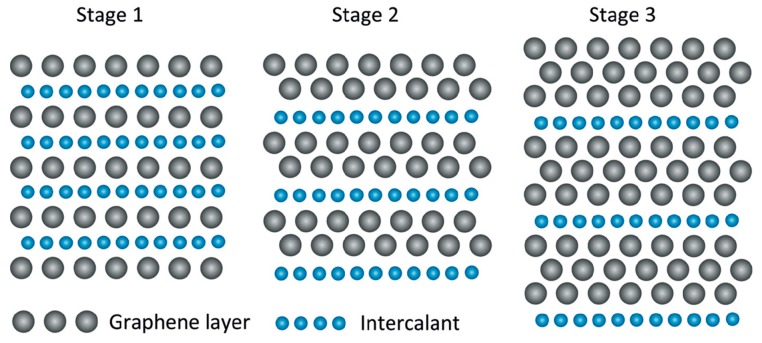
Schematic illustration of graphite intercalated compounds (GIC). Adapted with permission from [81]. Copyright © 2012 Elsevier.

GIC can be generally classified in terms of a “stage index *n*”, where *n* means the number of graphite layers between two adjacent intercalant layers. As shown in Figure 1, for example, GIC with stage of 1 indicates that 1 graphene layer is covered by adjacent intercalant layers. What should be mentioned here is that the intercalant layers can be more than 1 atom thick. Since most intercalants are inorganic, the formed GIC are generally classified according to the electrons that are donors or acceptors of intercalants. The most widely used donor intercalants are alkali metals [48]. Other donors like alkaline earth metals [56,57] and lanthanides [58,59,60] can also be used to synthesize donor GIC. When it comes to electron acceptor intercalants, a very large variety of compounds have been prepared using Lewis acid intercalants such as halogen [61], metal chlorides [50,51,62], bromides [63], fluorides [64] and oxyhalides [55], acidic oxides such as SO_3_, and strong Brønsted acids [65,66] such as H_2_SO_4_ or HNO_3_. The dominant method for synthesizing GIC is the two-zone vapour transport method [67,68,69]. Intercalation in intercalants that are molten [62,70] or in solution [71] can also obtain GIC. Apparently, the intercalation process is dominated by the donor–acceptor interaction between host graphite and guest intercalants. Another way to achieve intercalation is to utilize electrochemical reactions. The graphite can act as either an anodic electrode or cathodic electrode depending on the electrophile or nucleophile of intercalants [72,73,74,75,76]. It is worth mentioning that if the graphite was applied as an anode, the lithium ion can penetrate into the graphite layer and recombine with electrons in the intergallery to form stable intercalation compounds [77]. This process has been developed to commercialize lithium-ion batteries [78,79] and further improved in aluminum-based batteries [80] when an aluminum foil anode and ionic liquid electrolyte are used.

### 2.2. Organic Intercalating Compounds

#### 2.2.1. Electrochemical Methods

**Figure 2:**
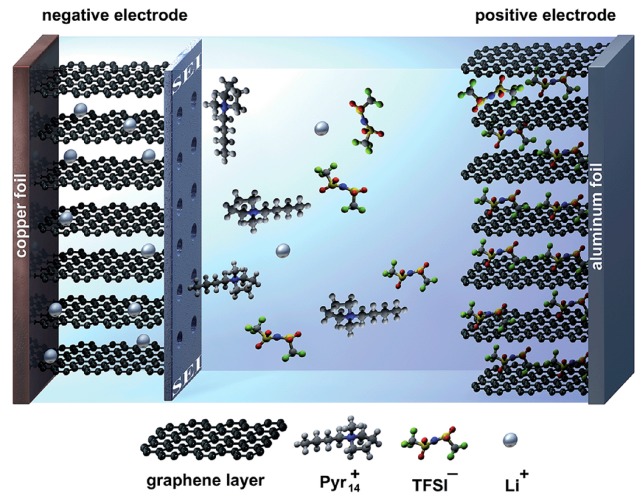
Schematic illustration of a dual-graphite cell with no effective solid electrolyte interphase layer at the graphite anode during the charge process. The negative graphite electrode suffers from exfoliation reactions caused by co-intercalation of the relatively large Pyr_14_^+^ cations [82]. Published by The Royal Society of Chemistry.

Similarly, organic molecules or ions can also achieve intercalation, but only a few studies have been reported. The intercalation of organic molecules by electrochemical methods is always regarded as a side effect of lithium-ion battery charging. Ionic liquids like *N*-butyl-*N*-methylpyrrolidinium bis(trifluoromethanesulfonyl)imide (Pyr_14_TFSI) or its smaller derivative, and other carbonates like propylene carbonate (PC), dimethyl sulphoxide (DMSO) and dimethylformamide (DMF)s are typically applied as an electrolyte for a lithium-ion battery. When charging, the *N*-butyl-*N*-methylpyrrolidinium cation (Pyr_14_^+^) [82,83] or PC [84] or other electrolyte molecules [85,86,87,88] can easily co-intercalate into the graphite anode with lithium ions. Besides the lithium-ion battery system, intercalation of organic molecules/ions by electrochemical methods mostly exist in the co-intercalation phenomenon with AlCl_4_^−^, PF_6_^−^, ClO_4_^−^ et al. [80,89,90,91]. Palermo et al. [91] reported that acetonitrile can co-intercalate with ClO_4_^−^, and ClO_4_^−^ and is indispensable in the intercalation process. This process involves intercalation of the large and negatively charged ClO_4_^−^ through grain boundaries or defect sites of a graphite anode, which favor the further penetration of the smaller, uncharged acetonitrile molecules. What should be mentioned here is that although most research focuses on organic molecules for co-intercalation, sporadic investigations indicate that organic ions can singly penetrate the interlayer of graphite in a special environment, for examples, dual-graphite cells [82,83] schemed in Figure 2. When Pyr_14_TFSI and lithium bis(trifluoromethanesulfonyl)imide (LiTFSI) were applied as an electrolyte, the graphite anode can be intercalated by bis(trifluoromethanesulfonyl)imide anion (TFSI^−^) individually in the charging process.

#### 2.2.2. Cation Exchange Methods

Cation exchange is another effective method for the intercalation of organic compound [92,93]. This idea follows a similar mechanism to the intercalation of montmorillonite [94], but unfortunately, no cation lives in the intergallery of pure graphite. Therefore, graphite should be pre-treated to ensure enough cation in its interlayers. Lerner et al. [95] used GIC as raw material, and the Na-ethylenediamine complex in the interlayers can be easily displaced by tetrabutylammonium ion (TBA^+^) in DMF through a cation-exchange reaction. Moreover, cation exchange can also perform in the electrochemical process. While Li^+^ have intercalated into the graphite cathode in charging, positively charged TBA^+^ can penetrate into the graphite lattice by cation exchange with the intercalated lithium ions [96]. However, electrodecomposition of the intercalated TBA^+^ appears in this reaction, and thus it is hard to obtain a stable TBA^+^ intercalated compound.

#### 2.2.3. Chemical Methods

Organic molecules can also directly intercalate into graphite layers by chemical methods, but this method always involve co-intercalation with alkali metal cations [71,97]. Metallic Li, Na, or K together with 1,2-diaminopropane (1,2-DAP) can realize co-intercalation with the protection of inert gas, but this process always takes a long time (1–3 days) [71]. The resulting compounds show different orientations of 1,2-DAP in the interlays, depending on the co-intercalated alkali metal.

However, the intercalation of pure organic molecules by chemical methods is far more difficult than co-intercalation with the help of alkali metals. Limited research has been done to successfully synthesize organic GIC using only graphite and organic intercalants. Although it is hard to form an organic layer in the graphite gallery, a limited number of organic molecules can still intercalate into graphite by π–π or cation–π intercalation between intercalants and graphite. Naphthalene, which consists of a fused pair of benzene rings, can penetrate into the edge of graphite, without further intercalation, acting as a “molecular wedge” [98]. This result was confirmed by the slight shift and obvious intensity decrease of the (002) plane of graphite in an X-ray diffraction (XRD) pattern. Similar results were obtained for the intercalation of cationic aniline (denoted as ANi^+^) [53] and caprolactam onium ion (denoted as CL^+^) [18], although the major driving force for intercalation is cation–π intercalation rather than π–π intercalation.

**Figure 3:**
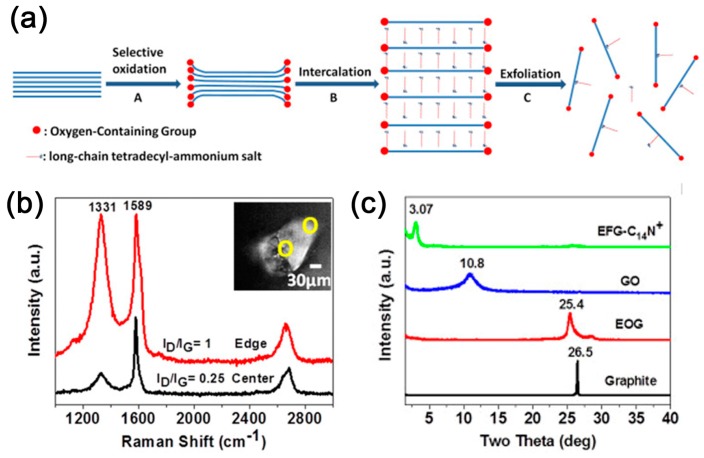
(**a**) Schematic illustration of the intercalation of edge-selective oxidized graphite (EOG); (**b**) micro-Raman spectra measured at the edge and on the basal plane of EOG; and (**c**) X-ray diffraction (XRD) of graphite, EOG, graphene oxide (GO) and EOG-C_14_N^+^ intercalated compound. Adapted with permission from [99]. Copyright © 2013 Springer Nature.

The intercalation of organic molecules into the graphite crystal is intrinsically impeded by the interlay’s Van der Waals interaction. Therefore, weakening of the inter-plane interaction would significantly facilitate the intercalation process. The most widely used method is oxidation of graphite. As schemed in Figure 3, natural graphite oxidized by low-concentration KMnO_4_ at relatively higher temperature can lead to edge-selectively oxidized graphite (EOG) with low-degree oxidation. Long-chain tetradecyl-ammonium cation (C_14_N^+^) can then spontaneously intercalate into graphite, forming an integrated C_14_N^+^ layer in the graphite gallery [99]; in other words, intercalation compounds. If there is a higher oxidation degree for graphite, it may transform into graphite oxide with a larger distance and weaker interaction between graphite planes, making it easier to capture more and larger molecules, for example, tetraalkylammonium ions (TAA^+^) [100], alcohol [101] or even polymers like poly(vinyl alcohol) (PVA) [102], poly(diallyldimethylammonium chloride) (PDDA) [103] and poly(vinyl acetate) (PVAc) [104] etc.

**Figure 4:**
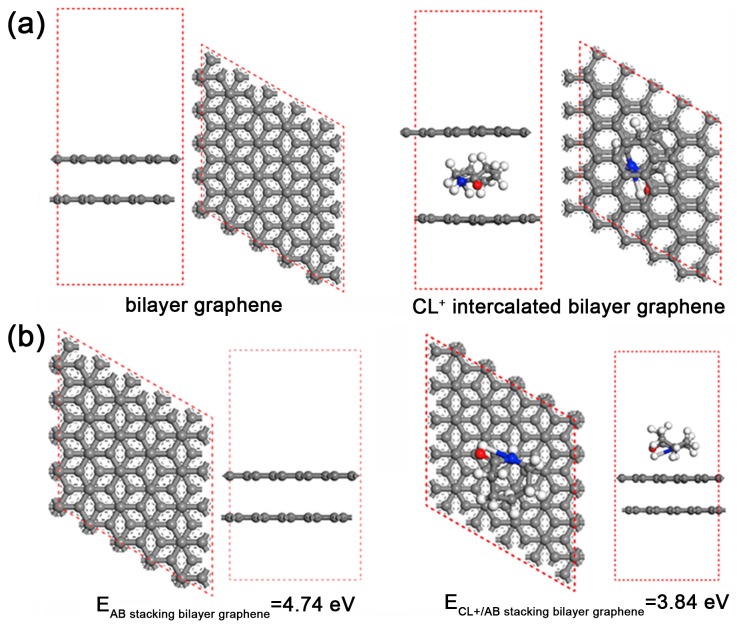
(**a**) The geometric structures of bilayer graphene and CL^+^ intercalated bilayer graphene; and (**b**) the calculated interlayer binding energy of the AB stacking bilayer graphene and CL^+^ absorbed bilayer graphene. Adapted with permission from [18]. Copyright © 2017 Elsevier.

Despite the difficultly in forming an organic layer, the intercalation of special molecules into expanded graphite (EG) or natural graphite has been confirmed, as mentioned above. Basically, the driving force for intercalation was firstly due to π–π intercalation between intercalants and graphite. This idea is proved by the fact that naphthalene and aniline (ANi), both of which possess benzene rings, can intercalate into graphite layers [53,98]. As shown in Figure 4, first-principle simulation of the intercalation of ANi molecule into bilayer graphene was performed by Zhou et al. [53]. The positive formation energy of 2.01 eV proved its energetically favorable reaction. Meanwhile, it was noticed that the cationic ANi would be easier to intercalate into the graphite layers as ANi^+^ obtains higher formation energy of 2.81 eV, and experimental data further confirmed the simulation results. It seems that the cation–π interaction between the intercalary cation and the graphite interlayer of the π-electron is another important force for intercalation. This theory was soon authenticated by the further study on the intercalation of CL^+^ [18]. By comntrast with ANi^+^, CL^+^ do not have a benzene-like structure, and thus there is no π–π intercalation between CL^+^ and graphite. Consequently, the intercalation force is almost all attributed to the cation–π interaction. Moreover, research also indicates that the adsorption of cation on the graphite surface can significantly decrease the interaction between the graphite layers [18], facilitating the succeeding intercalation of organic cation.

## 3. Polymerization in the Interlayers of Graphite

### 3.1. Intercalation Polymerization Methods

**Figure 5:**
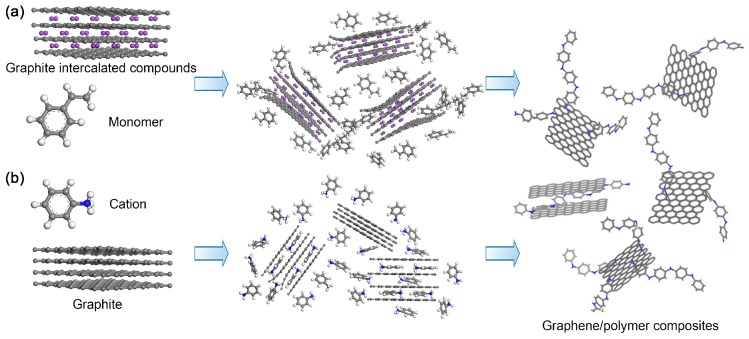
Schematic of the two kinds of polymerization in the interlayer of graphite: (**a**) polymerization initiated by pre-intercalated compounds; and (**b**) polymerization initiated after intercalation of monomers (in situ intercalation polymerization).

Polymerization in the interlayers of graphite can be generally divided into two strategies as illustrated in Figure 5: polymerization initiated by pre-intercalated compounds and polymerization initiated after the intercalation of monomers (in situ intercalation polymerization).

#### 3.1.1. Polymerization Initiated by Pre-Intercalated Compounds

For this situation, GIC is always used as pre-intercalated compounds. When graphite is intercalated by alkali metals, an electron cloud of the alkali metal tends to migrate to graphite, thus forming an ionic compound [55]. Then, anionic polymerization of vinyl or epoxide monomers can be initiated by the negatively charged graphite layer of the alkali metal–GIC [105,106]. However, limited by the interlayer distance of GIC, monomers are hard to absorb into the interlayer of graphite in solution for further polymerization [107]. Instead, unsaturated hydrocarbon vapor such as styrene or isoprene were used to penetrate the interlayer galleries of potassium intercalated graphite, and then these underwent anionic polymerization, leading to the gradual expansion of the distance between graphite layers and the final exfoliation of graphite nanosheets [108,109]. It should be noted that the stage of alkali metal–GIC seems to be important for controlling the intercalation polymerization. For example, when KC_24_ (stage 2 potassium intercalated graphite) is used as the initiator, the reaction rate of intercalation polymerization can be several times faster than that of KC_8_ (stage 1 potassium intercalated graphite) [108]. However, KC_8_ exhibits much more effective exfoliation of graphite layers, while the products obtained from higher-stage GIC are mixed with un-exfoliated graphite [105].

**Figure 6:**
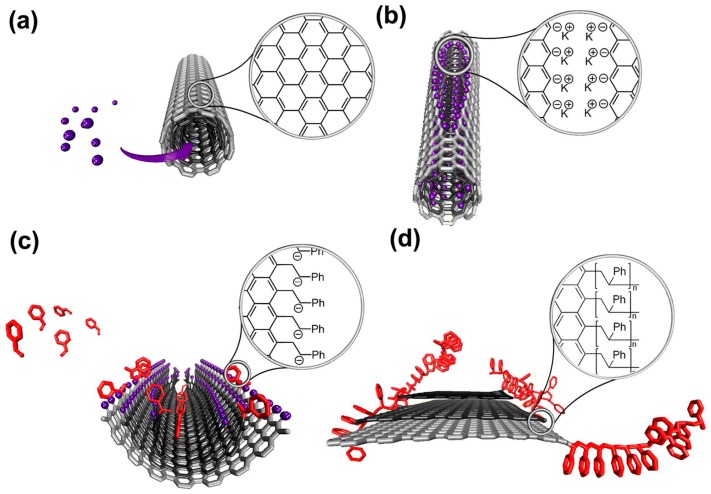
Reaction scheme of multiwalled carbon nanotubes (MWCNTs) unzipping and edge-selective in situ polymerization of vinyl monomers. (**a**) Intercalation of MWCNTs by potassium naphthalenide; (**b**) formation of longitudinal fissure in the walls; (**c**) polymerization of styrene assists in exfoliation of MWCNTs; (**d**) polymer functionalized GNRs. Adapted with permission from [110]. Copyright © 2013 American Chemical Society.

**Figure 7:**
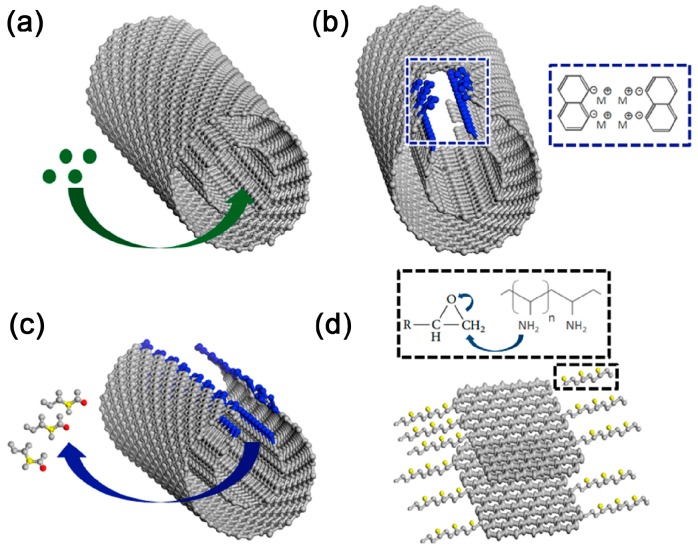
Reaction scheme of unzipping and edge-functioned MWCNTs for initiating polymerization of the epoxy resin. (**a**) intercalation of MWCNTs by alkali metal; (**b**) longitudinal unzipping and formation of carbanions, stabilized by cation; (**c**) in situ functionalization of unzipped MWCNTs by *N*-vinylformamide; (**d**) edge-functioned MWCNTs formation upon *N*-vinylformamide hydrolysis. The polymerization reaction is marked by dashed box. Adapted with permission from [106]. Copyright © 2016 Elsevier.

Besides the intercalation polymerization initiated by alkali metal–GIC, some interesting work has been reported to synthesize polymer functionalized graphene nanoribbons (GNRs) using multiwalled carbon nanotubes (MWCNTs) as raw material [110]. In an analogy to the intercalation chemistry of graphite, the intercalation of potassium vapor or solvent-stabilized potassium cations into MWCNTs can lead to an expansion of the d-space between MWCNT layers, causing the MWCNTs to partially or fully split [111,112,113]. Thus, the fissures are functioned with aryl anions and their associated metal cations and converted into edge-negatively charged macroinitiators for the subsequent anionic polymerization of vinyl monomers [110]. This strategy can be described in Figure 6. Furthermore, the active carboanionic edge of unzipped MWCNTs can be further functioned by *N*-vinylformamide to act as nucleophilic agents and initiate a polymerization of epoxy resin (Figure 7) [106]. Thus, GNR functioned with different kinds of polymers can be synthesized following this idea [114,115,116]. Since the active carboanionic site mostly appears at the edges of GNR, it would always result in site-selective polymerization. Therefore, this strategy leads to polymer functionalized edges of graphene nanoribbon, but the basal planes can still remain sp^2^-hybridized carbon [110].

#### 3.1.2. In Situ Intercalation Polymerization

**Figure 8:**
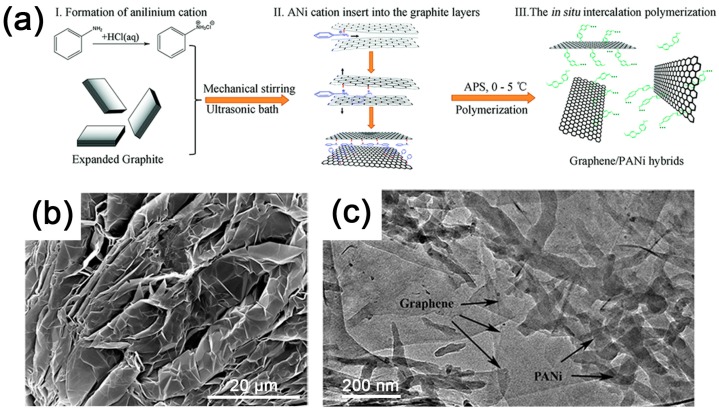
(**a**) Schematic for the in situ intercalation polymerization of ANi^+^ into EG to synthesize graphene/polyaniline hybrids; (**b**) scanning electron microscope (SEM) image of expanded graphite; and (**c**) transmission electron microscope (TEM) image of graphene/polyaniline hybrids obtained by in situ intercalation polymerization. Adapted with permission from [53]. Copyright © 2014 Royal Society of Chemistry.

As mentioned, some kinds of organic molecules can intercalate into graphite by π–π or cation–π intercalation between intercalants and graphite interlayers. Although a limited amount of molecules can penetrate into the interlayer of graphite, these polymerizable monomers can be initiated by subsequently added initiators. Zhou et al. performed the polymerization of aniline confined in graphite layers, resulting in graphene/polyaniline (PANi) hybrids by a one-step in situ intercalation polymerization [53]. An alogous method was then applied to prepare polypyrrole (PPy) or polyamide-6 (PA-6)/graphite nanoflake composites, confirming the universality of in situ intercalation polymerization [18,117]. This strategy is summarized in Figure 8. Monomer cations absorb on the surface of graphite to decrease the interaction between graphite layers, which facilities the following intercalation of monomer cations by π–π or cation–π intercalation. As more cationic complexes insert into the layers, the graphite interlayer space turns to a larger space and thus further weakens the intercalation between interlaminations. After initiating the polymerization, monomer cations confined in graphite interlayers grow into polymer chains gradually. A large amount of heat would be generated in this process, involving the movement of long-chain molecules. These effects lead to a violent separation of graphite and exfoliate into graphene. Furthermore, the exfoliated graphene is pasted and stabilized by the onsite synthesized polymer molecules to prevent its agglomeration.

Since the interlayer distance of graphite is 3.35 Å, it can be thought as a natural nanoreactor, and in situ intercalation polymerization performed in the graphite interlayers can be recognized as a typical 2D-confined polymerization. Moreover, sp^2^-hybridized carbon in graphite provides abundant π-electrons, leading to a special 2D electron-rich confined space. Polymer synthesized in such a unique nanoscale-confined space is partitioned from that of the surrounding bulk space. In situ polymerization in the interlayer of graphite results in the hybridization of graphene/polymer induced by the nano-confined effect and electron interaction, which may further influence the band structure of hybrids [53,117,118]. In addition, a nano-confined space always causes geometric conformational transformation or orientation of confined molecules [71], which might be used for the further study of molecular structure.

**Figure 9:**
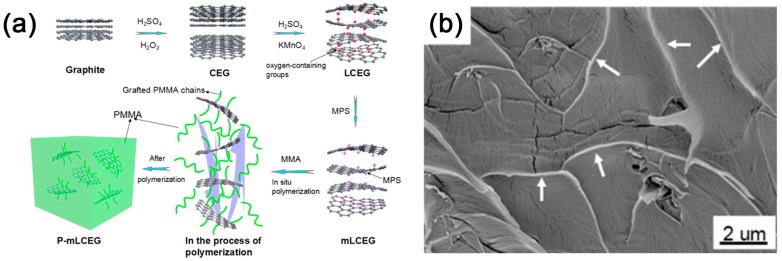
(**a**) Schematic of the preparation of polymethyl methacrylate (PMMA)/graphene composites by interlayer polymerization; and (**b**) SEM image of freeze-fractured cross sections of composites. Adapted with permission from [119]. Copyright © 2017 American Chemical Society.

Owing to its larger interlayer distance and functioned oxygen-containing group, graphite oxide can be more easily intercalated than graphite by not only the cationic complex but also molecules like vinyl alcohol [107], vinyl acetate [104] and methyl methacrylate [119] for interlayer polymerization. Sandwich-like polymer/graphene oxide composites with highly crumpled and intercalated structures can be obtained by the in situ interlayer polymerization [107,119,120]. The extraordinary crumpled structure might be attributed to the interlayer chain movement and hybrid interactions between the polymer and graphene oxide. Besides weakening of interlayer interaction, some research indicates that the surface wettability of graphite to monomers is another critical factor for the exfoliation of graphene [119]. Chemical expanded graphite (CEG) is used for further oxidization to introduce oxygen functional groups on the graphite surface. Benefiting from its open and highly surface-accessible pore structures, diffusion resistance of the oxidizer in the interlayers of CEG significantly reduces [121]. The two-stage oxidization (as illustrated in Figure 9a) results in the spatially uniform oxidization of graphite layers (Figure 9b) which is different from traditional graphite oxide functioned mostly in the peripheral region [122,123]. In this way, the wetting capability of CEG to monomers can be improved by the uniformly grafted oxygen functional groups, and finally leads to spontaneously and uniform exfoliation of CEG into single- and few-layer graphene in graphene/polymer composites during the interlayer polymerization.

### 3.2. Characterization of Intercalation Polymerization

**Figure 10:**
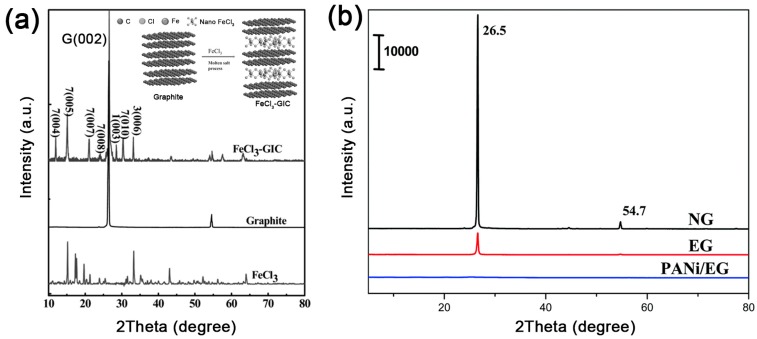
(**a**) XRD patterns of graphite, FeCl_3_ and FeCl_3_-GIC; insert is the schematic of graphite and FeCl_3_-GIC. Adapted with permission from [62]. Copyright © 2014 Royal Society of Chemistry. (**b**) XRD patterns of natural graphite, expanded graphite (EG) and graphene/polyaniline (PANi)/EG hybrids synthesized by intercalation polymerization. Adapted with permission from [53]. Copyright © 2014 Wiley Online Library.

For the characterization of intercalation polymerization, the primary consideration is to focus on the intercalation and exfoliation of graphite, and XRD is the most important test. The XRD pattern of graphite always exhibits sharp characteristic diffraction peaks at 2θ = 26.5° (*d* = 3.35 Å), which are assigned to the (002) plane of graphite. The interlayer distance will be enlarged if graphite is intercalated by a guest molecule, leading to intensity decrease or disappearance of this peak. Instead, new diffraction peaks corresponding to the changed interlayer distance may appear as shown in Figure 10. Once intercalation polymerization achieves the exfoliation of graphene, these peaks will disappear due to the separation of graphite layers. Therefore, the XRD pattern can be used to effectively analyze the intercalation and exfoliation of graphite.

**Figure 11:**
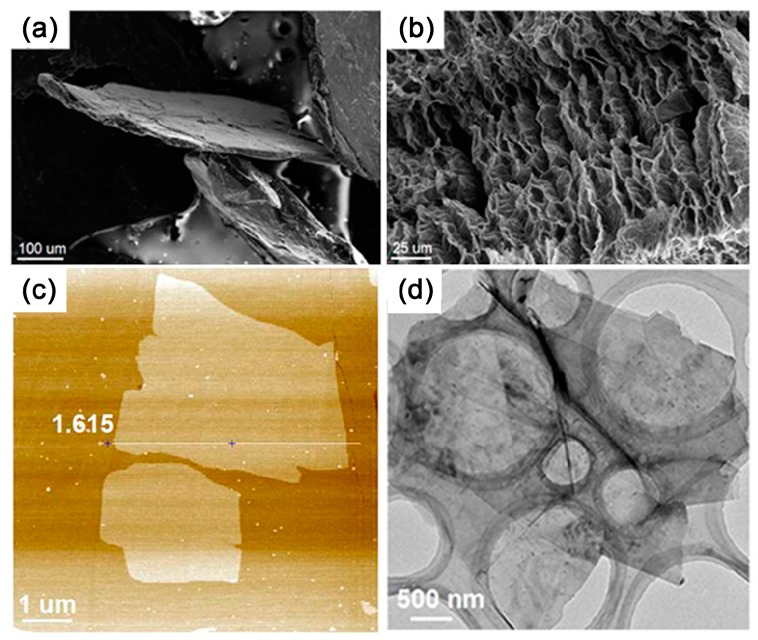
SEM images of (**a**) natural graphite and (**b**) expanded graphite; (**c**) atomic force microscope (AFM) image and (**d**) TEM image of PMMA/graphene composites. Adapted with permission from [119]. Copyright © 2017 American Chemical Society.

Exfoliated graphene can also be distinguished by morphology characterization using a scanning electron microscope (SEM), transmission electron microscope (TEM) and atomic force microscope (AFM) etc. Highly-stacked natural graphite or worm-like expanded graphite are significantly different from exfoliated single- and few-layer graphene, as depicted in Figure 11. Furthermore, high-resolution TEM and AFM can be helpful in confirming the number of graphene layers. It must be noted that sometimes the number of graphene layers calculated from thickness are not accurate due to the coated polymer on the graphene.

**Figure 12:**
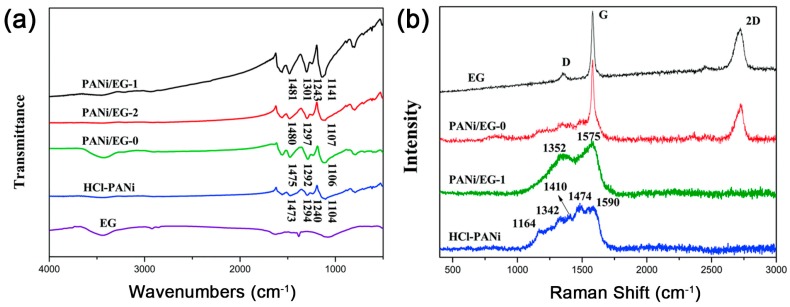
(**a**) Fourier-transform–infrared (FT–IR) and (**b**) Raman spectra of expanded graphite (EG), PANi and PANi/EG composites. (PANi/EG grinding mixture was denoted as PANi/EG-0, the intercalation polymerization and in situ polymerization of ANi^+^ into 1 wt % EG was denoted as PANi/EG-1 and PANi/EG-2, respectively.) Adapted with permission from [53].Copyright © 2014 Royal Society of Chemistry.

As the intercalation polymerization goes on in a typical 2D-confined space, the structure of graphene may change due to the hybridizing interactions between exfoliated graphene and synthesized polymer. Therefore, some forms of structural characterization can also be applied to analyse intercalation polymerization such as Fourier-transform–infrared (FT–IR) spectra and laser Raman spectroscopy. For example, the interaction between the N-atom in PANi and π-electrons in graphene leads to the blue shifts of C–N, C=N stretching vibrations (Figure 12a). Meanwhile, the exfoliation and hybridization of graphene influence the π-electron cloud in graphite, resulting in overlapping of the D band (at 1350 cm^−1^) and G band (at 1580 cm^−1^), and the disappearing of the 2D band (at 2700 cm^−1^) in the Raman spectra (Figure 12b) [53].

Besides the above-mentioned methods, many other characterizations have been used to study the intercalation polymerization and synthesized composites, such as X-ray photoelectron spectroscopy (XPS), differential scanning calorimetry (DSC) and polarized optical microscopy (POM) etc. [18,119] However, some fundamental research, for example that on intercalation efficiency, are still challenging, and require further study. With the development of in situ characterization and theoretical simulation, a better understanding of intercalation polymerization can be achieved.

### 3.3. Influence Factors on Intercalation Polymerization

As presented above, intercalating molecules/ions/clusters into graphite, and polymerization in the graphite interlayers, are the key points for intercalation polymerization. There are many factors affecting this process. Therefore, based on literature results, we mainly review the influencing factors on intercalation polymerization from three aspects, i.e., the source of graphene, intercalant species, and process parameters of intercalation polymerization.

#### 3.3.1. Source of Graphene

The source of graphene in resulting graphene/polymer composites is important in the intercalation polymerization. It can be divided into natural graphite, expanded graphite, modified graphite and carbon nanotube. Because of the differences in structure, their performances in intercalation and exfoliation are also different.

**Figure 13:**
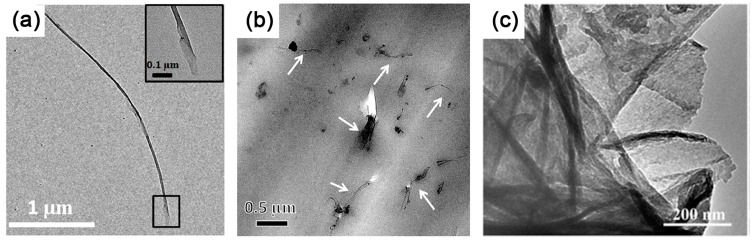
TEM images of (**a**) partially unzipped MWCNTs, inset is the unzipped layer; and (**b**) graphene nanoribbon (GNR)/epoxy nanocomposites. Adapted with permission from [106]. Copyright © 2016 Elsevier. (**c**) Polypyrrole (PPy)/GO synthesized by intercalation polymerization. Adapted with permission from [120]. Copyright © 2016 Elsevier.

Natural graphite with a complete crystal structure and large planes are the first choice for preparing high-quality graphene. Most research into traditional GIC used NG as raw materials. As mentioned above, NG can be fully intercalated by alkali metal, but few studies have achieved the intercalation of organic monomers [55]. That might be due to its highly stacked layers. Thus, until now NG has only been used as an initiator for anionic polymerization after the intercalation of alkali metal. However, only thick graphite flakes are exfoliated in related reports, indicating an insufficient contact between polymerizable monomers and the initiating segment of GIC [105,108,109]. It seems that monomers can only contact the edge of the GIC without further penetrating into the interlayer galleries, leading to limited exfoliation. Potassium intercalated MWCNTs are in a similar situation when applied in intercalation polymerization. While intercalation of MWCNTs leads to a partial or full split, monomers can only polymerize at the edges of fissures without further intercalating [106,110]. Actually, when MWCNTs are used for polymerization, as shown in Figure 13a,b, the size of GNR in the resulting composites is quite small and limited by the superficial area of pristine MWCNTs [106,115].

The intercalation of organic monomers is impeded by the highly-stacking layers due to strong interlayer interaction of natural graphite. The stacked layers of NG also lead to a limited area of accessible surface for monomers. Therefore, EG with an open, highly surface-accessible pore structure (Figure 11a,b) is the best substitute for NG, which facilitates the access and intercalation of monomers [53,117]. As more monomers are able to absorb on the surface of EG due to the worm-like structure, single- or few-layered graphene with large scale (Figure 8c) can be effectively exfoliated by the subsequent polymerization [53]. However, the exfoliation of EG is insufficient at relatively higher filler loading (more than 4 wt %), indicating the limitation of utilizing the physical structure of graphite.

Comparing with NG and EG, GtO and CEG possess not only larger interlayer distance due to weakened inter-plane interaction, but also abundant functional groups including hydroxyl, carboxyl and grafted molecules. These functional groups induce strong interaction between monomers and graphite layers, thus making for effective intercalation and exfoliation (Figure 13c) [119,120]. Furthermore, the modification of graphite significantly improves the surface wettability of graphite layers to monomers, resulting in the spontaneous exfoliation of graphene. It seems that reasonable modification of graphite can be helpful in intercalation polymerization together with the highly accessible surface area of graphite layers, which inspire us to tune the structure of graphite for more efficient intercalation polymerization.

#### 3.3.2. Intercalant Species

The choosing of intercalant is another key factor for graphite intercalation. Traditional intercalants for synthesising GIC have been systematically reviewed in ref. [55], but the organic intercalants have not been discussed before. In order for the one-component organic molecules to intercalate, their structure should be carefully considered. Intercalation of naphthalene or aniline molecules infers that π–π interactions can be utilized in this process [53,98]. However, the incomplete intercalation indicates that π–π interactions are not strong enough for sufficient intercalation. Benefiting from the strong cation–π interactions, aniline cation exhibits a more pronounced effect in intercalation and exfoliation [53]. Moreover, the successful intercalation of pyrrole cation or caprolactam onium ion further confirms the contribution of a positive charge [18,117]. Thus we can say that the intercalative process is dominated by the strong cation–π interactions between monomers and graphite, and π–π interactions may also help this process.

#### 3.3.3. Process Parameters of Intercalation Polymerization

**Figure 14:**
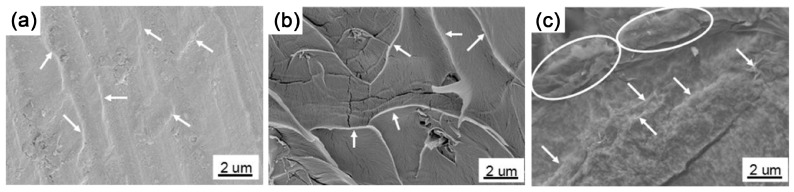
SEM image of freeze-fractured cross sections of PMMA/chemical expanded graphite (CEG) with CEG contents of (**a**) 1 wt %, (**b**) 4 wt %, (**c**) 10 wt %; graphene sheets are denoted by the arrows, and the ovals indicate aggregations of graphene sheets. Adapted with permission from [119]. Copyright © 2017 American Chemical Society.

Feeding a ratio of monomers to graphite can significantly influence the exfoliation and dispersion of graphene in the polymer matrix. Because of its poor ability in dissolution, the addition of graphite is always less than 1 wt % of monomers, otherwise exfoliated graphene would be difficult to homogeneously disperse in the matrix [117]. But for hydrophilic CEG or GtO, their content can even be increased up to 10 wt % with only a few aggregations, as shown in Figure 14 [119].

Moreover, ultrasonication is necessary to help the monomers intercalating into the interlayers of graphite. With the ultrasonication-assisted intercalation, worm-like EG or stacked GtO can be separated and dispersed into flakes [53]. However, it is easy to understand that violent ultrasonication may break the complete graphite layers into small fragments. With short-duration ultrasonic exfoliation, large GO flakes (lateral size of 10–20 µm) can be obtained. Long-duration ultrasonic also results in flakes smaller than 1 µm, with more than 75% of them having a size in the range 0.1–0.4 µm [124]. Thus, a mild and reasonable power of ultrasonication is of importance in the intercalation process, which facilities high-efficient exfoliation in the polymerization.

## 4. Application of Graphene/Polymer Composites

Intercalation polymerization provides a new method for synthesizing graphene/polymer composites. Polymerization conducted in the 2D-confined space of graphite layers leads to graphene and polymer hybrids which can be easily distinguished from general polymers synthesized in normal environment. Strong hybridization interaction between polymer molecules and graphene can induce some amazing performance change. In this section, some emerging applications of graphene/polymer composites synthesized by intercalation polymerization are reviewed, including electrical conductivity, electromagnetic absorption, mechanical properties and thermal conductivity.

### 4.1. Electrical Conductivity

Graphene is widely used as nanofiller for improving the electrical conductivity of polymers and decreasing the percolation threshold, because of its large specific surface area and extraordinary electrical property. But contrary to original intentions, the agglomerate of graphene sheets in polymer composites during processing always inhibits the expected effects. In situ polymerization conducted in the interlayer of graphite not only exfoliates graphene layers, but also isolate layers by onsite synthesized polymer. For the PMMA/graphene composite synthesized by intercalation polymerization with the addition of 1.5 wt % of CEG, electrical conductivity increases about 12 orders of magnitude to 1.63 × 10^−2^ S/m [119]. This value is far beyond the percolation threshold, implying the good dispersion of exfoliated graphene in composites. Even more astonishing, a PMMA/graphene composite with an extremely high electrical conductivity of 1719 S/m can be obtained when 10 wt % of CEG was used in polymerization, which is one of the highest values reported for graphene/polymer composites as compared in Table 1.

However, interesting results are reported when conducting polymers were used for interlayer polymerization. Polyaniline/graphene hybrids synthesized by in situ intercalation polymerization display obvious decrease in electrical conductivity as compared to those of HCl doped polyaniline or expanded graphite [53]. This can mostly be attributed to the hybridizing intercalation between polyaniline molecule and graphene. While the interlayer of graphite acts as nanoreactors, the strong confined effect would occur during the confined polymerization, which behaves as electron cloud migration between graphene and polymer molecules. The hybridizing intercalation, on the one hand, reduces the doping degree of polyaniline, leading to lower carrier concentration, and, on the other hand, affects the conjugated system in graphene. Furthermore, π–π staking might also exist in graphene/polyaniline hybrids. Taken together, the electrical conductivity of the hybrids exhibits an unusual decrease when compared to pure polyaniline or expanded graphite.

### 4.2. Electromagnetic Wave Absorption

While digital devices and rapid development of radar detecting technology change our lifestyle, the electromagnetic waves (EM) generated also lead to the grim problem of EM interference. Thus the protection and shielding of electromagnetic radiation has been widely considered as a serious problem, and the microwave absorbing materials is desperately desired by society. As is known, impedance matching and EM-wave attenuation in the interior of materials are two principles for promoting EM-wave absorption. The former ensures as little reflection as possible at the surface of materials, and the latter leads to energy dissipation of the EM wave. Therefore, synergistic effects of the dielectric loss and magnetic loss are important for promoting EM absorption.

**Figure 15:**
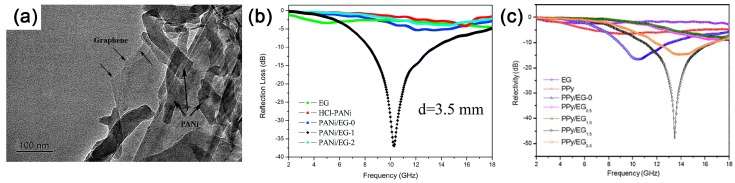
(**a**) TEM image of PANi/EG hybrids hybrids synthesized by intercalation polymerization of ANi^+^ into 1 wt % EG; and (**b**) the calculated RL in the frequency range of 2–18 GHz (PANi/EG grinding mixture was denoted as PANi/EG-0, the intercalation polymerization and in situ polymerization of ANi^+^ into 1 wt % EG was denoted as PANi/EG-1 and PANi/EG-2, respectively). Adapted with permission from [53]. Copyright © 2014 Royal Society of Chemistry. (**c**) Calculated RL of PPy/EG with a thickness of 2.7 mm in the frequency range of 2–18 GHz (hybrids with different addition of EG were denoted as PPy/EG*_x_*, where *x* = 0, 0.5, 1.0, 1.5, 2.0 wt %). Adapted with permission from [117]. Copyright © 2015 Elsevier.

Intercalation polymerization has brought some obvious change in physical parameters for graphene/conductive polymers. For example, the conductivity and permittivity of the hybrids exhibit extraordinary change as compared with pure conductive polymers or graphite. A much better impedance match can be obtained for graphene/polyaniline hybrids synthesized by intercalation polymerization, facilitating the improvement of microwave absorption [53]. Besides, defects and hybridizing points induced by hybridizing interaction between polyaniline and graphene act as an extra polarization center and cause additional relaxation. As shown in Figure 15, the resulting hybrids show significant enhancement in microwave absorption, and the minimum reflection loss (RL) reached −36.9 dB with a thickness of 3.5 mm. Moreover, absorption bandwidth with RL below −10 dB is in the frequency range of 5–18 GHz, depicting a broad frequency band for the application. Furthermore, based on intercalation polymerization, our group has also developed other similar works such as graphene/polypyrrole or graphene oxide/polypyrrole hybrids for microwave absorption [117,120].

**Figure 16:**
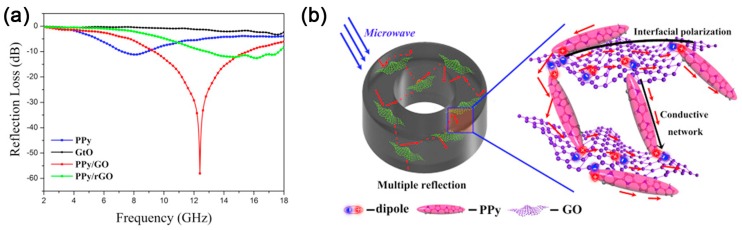
(**a**) The reflection loss (RL) of the samples with a thickness of 2.96 mm; and (**b**) schematics of electromagnetic waves (EM) loss-enhancement mechanism of PPy/GO. Adapted with permission from [120]. Copyright © 2016 by the Elsevier.

Among these hybrids, PPy/GO exhibits the best result for microwave absorption. The minimum RL reaches −58.1 dB at 12.4 GHz with a thickness of 2.96 mm, and a wide broad bandwidth (< −10 dB) of 6.2 GHz (Figure 16a) indicates its extraordinary performance among different microwave-absorbing materials [120]. For graphene/conductive polymer composites, their EM loss mainly comes from dielectric loss with almost no magnetic response. Benefiting from the strong hybridization effect, the interaction between –NH in PPy and –CO in GO introduce new unsymmetrical centers, which results in additional charge rearrangement and orbital hybridization due to electric dipole polarization. In addition, crumpled structures of PPy/GO (as shown in Figure 13c) would lead to more interfacial losses or relaxations at a higher frequency. The mechanism for the dielectric loss enhancement of PPy/GO composite is illustrated in Figure 16b. Recent work on microwave absorption of polymer composites is summarized in Table 2. It can be seen that intercalation polymerization plays a key role in the polymer composites to improve their performance in microwave absorption.

### 4.3. Mechanical Properties

**Figure 17:**
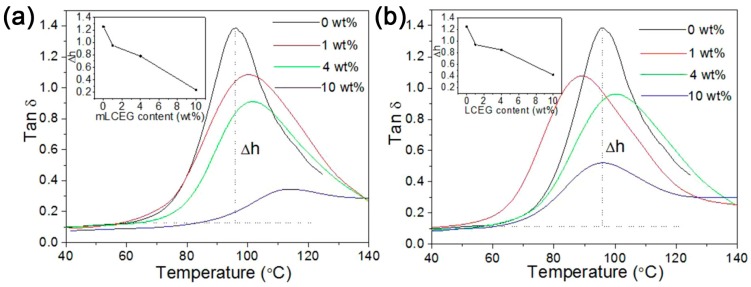
(**a**) Storage modulus; and (**b**) damping loss of PMMA/chemical expanded graphite (CEG) composites with different filler contents as a function of temperature. Adapted with permission from [119]. Copyright © 2017 American Chemical Society.

The mechanical properties of composites are worth expecting because of the homogeneous disperse of graphene and the strong interfacial interactions induced by in situ intercalation. When GtO is intercalated and exfoliated, the tensile strength of PVA increases from 42.3 MPa of pure PVA to 50.8 MPa with only 0.04 wt % GtO loading, and Young’s modulus increases from 1477 to 2123 MPa [107]. The significant improvement of mechanical properties at such low loading of GO can be due to the uniform dispersion of exfoliated GO, the aligned GO parallel to the film and the hydrogen bonding interaction between GO and polymer chains. But, limited by the initial strength of a dilapidated GO plane and the efficiency of intercalation polymerization, the mechanical properties of PVA are difficult to improve further. Thus, stronger interfacial interactions between graphene planes and polymer matrix are expected. Therefore, uniform oxidized graphite layers are functioned by introducing polymerizable C=C bonds on the graphene surface, ensuring polymer molecules covalent grafting onto graphene in subsequent interlayer polymerization, as shown in Figure 17 [119].

Covalent bonding between polymer chains and graphene planes leads to better interfacial interaction, cooperating with the good dispersion of graphene, composites exhibit a 3-fold increase in the storage modulus with 10 wt % functioned CEG [119]. As summarized in Table 3, the intercalation polymerization significantly improves the mechanical properties of composites when compared to other synthesis methods. Furthermore, gradually decreasing transition temperature and decreasing of damping loss indicates a typical restricted relaxation behavior and effective interface load transfer, which is reasonably related to the modified in situ intercalation polymerization.

### 4.4. Thermal Conductivity

**Figure 18:**
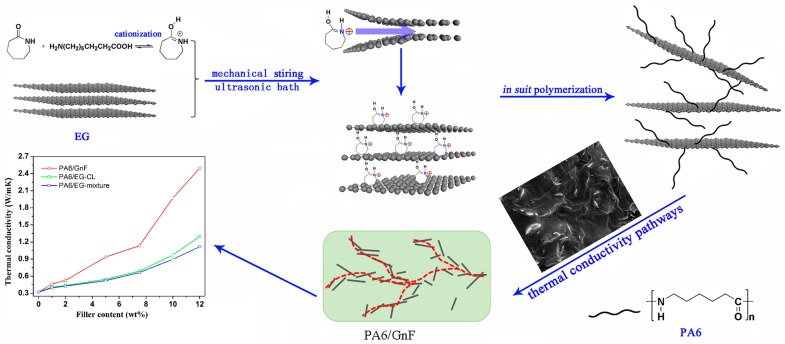
Schematic for the in situ intercalation polymerization of CL^+^ into EG to synthesize polyamide-6/graphite nanoflakes composites. Adapted with permission from [18]. Copyright © 2017 Elsevier.

Since most polymers exhibit poor ability in conducting heat flow, graphene has long been expected to promote their thermal conductivity (TC). Similar to electrical conductivity, the dispersion of graphene in the polymer matrix is one of the key points for higher thermal conductivity. Thus, in situ intercalation polymer can be a useful method for fabricating polymers with high thermal conductivities. As depicted in Figure 18, polyamide-6/graphite nanoflakes synthesized by intercalation polymerization exhibits significant thermal conductive improvement to 2.49 W/(m·K) with 12 wt % EG loading, as that of pure polyamide-6 is only 0.32 W/(m·K) [18,142]. Compared with composites prepared by in situ polymerization or melt mixing with EG, intercalation polymerization results in not only better dispersion of graphite nanoflake but also better interfacial connections. Generally, better compatibility always means a better phonon match between EG and the polymer matrix, further decreasing the thermal interface resistance and improving the percolation. Moreover, the thermal conductivity of PA-6 composites synthesized by the intercalation polymerization is much higher than that of most reported graphene/polymer composites (Table 4). Therefore, in situ intercalation polymerization provides a good idea for constructing highly efficient thermal conductive pathways within the matrix network.

### 4.5. Other Applications

**Figure 19:**
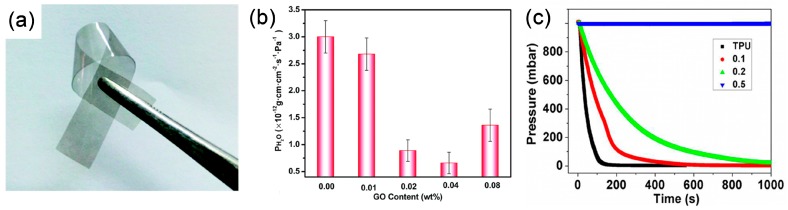
(**a**) Digital image of poly(vinyl alcohol (PVA)/GO composites film synthesized by intercalation polymerization; and (**b**) its water vapor permeability. Adapted with permission from [107]. Copyright © 2016 Royal Society of Chemistry. (**c**) Pressure drop curves of thermoplastic polyurethane (TPU)/GNR composites. Adapted with permission from [115]. Copyright © 2013 by the American Chemical Society.

Except for the above applications, graphene/polymer composites synthesized by intercalation polymerization have also been used in other fields like sensing, electrochemical supercapacitor and gas barriers. For examples, PVA/GO synthesized by intercalation polymerization can form an optically transparent, flexible film with much lower water vapor permeability than neat PVA, as shown in Figure 19a,b [107]. Similar results are reported for thermoplastic polyurethane (TPU)/GNR composites. Nitrogen gas effective diffusivity decreased by 3 orders of magnitude with only 0.5 wt % GNRs (Figure 19c) [115]. Some other applications of synthesized graphene/polymer composites are summarized in Table 5. Although a few researches, these works give a sight for expanding the application fields of intercalation polymerization.

## 5. Conclusions and Outlook

Based on the above generalizations about intercalation polymerization, it can be concluded that the intercalation chemistry of graphite and subsequent interlayer polymerization have attracted increasing attention, and research of intercalation polymerization and the resulting composites has indeed become attractive. The presented review has highlighted recent developments relating to intercalation, polymerization and the performance of the as-synthesized graphene/polymer composites.

For intercalation polymerization, what is important is the interaction between organic monomers and graphite interlayers. If the interaction is not strong enough, monomers cannot penetrate into the deep intergallery for sufficient exfoliation, which leads to only thick graphite flakes or edge-functioned layers. In situ intercalation polymerization successfully disperses graphene in synthesized polymer composites. However, the intercalation efficiency of monomers is still too low to form GIC, thus limiting the content of graphene in the matrix. Moreover, once organic monomer-GIC is successfully synthesized, the layer number of exfoliated graphene will be theoretically controllable. Therefore, improving the intercalation efficiency becomes a serious issue for intercalation polymerization, and tuning the interaction between monomers and graphite can be an effective way of doing this. What we can do to tune the interaction is to carefully design the structure of intercalative monomers and graphite. Cation–π interactions play an essential role in the intercalation process, and therefore cationic monomers or oxidized graphite achieve a better intercalation effect. If a conjugated structure exists in intercalants, π–π interactions may also assist the intercalation process. The oxidation and modification of graphite can significantly reduce the resistance of intercalation and exfoliation, and the introduced active sites facilitate the functional applications of composites. However, traditional methods prefer to attack the carbon atoms in the peripheral region, leading to inhomogeneous distribution of functional groups. In recent years, controllable and spatially uniform oxidation has been achieved using K_2_FeO_4_ or H_2_O_2_ [43,44]. These results inspire us to comprehensive consider when graphite oxide or modified graphite are used in intercalation polymerization. For example, the slightly but uniformly oxidized graphite achieve fully intercalation, spontaneous exfoliation and homogeneously dispersed graphene, thus leading to highly conductive and mechanically strong polymer composites [119]. The graphite oxide with a high degree of oxidation also improves the EM absorption of PPy [120].

Recently, graphene/polymer composites synthesized by intercalation polymerization have exhibited a significant improvement of performance in various fields. However, some related fundamental scientific issues should be studied. For instance, it is important to understand the structural evolution of polymers during polymerizing in the 2D space of the graphite interlayers. Thereafter, we can reveal the interaction mechanism between graphene and polymer molecules in the process of intercalation polymerization, which may aid in the further molecular regulation and functional design of polymer materials. It is believed that intercalation polymerization will offer a bright future in the field of the synthesis and application of graphene/polymer composites.

## Figures and Tables

**Table 1:** Comprehensive comparison of the electrical conductivity of graphene/polymer composites.

Material	Synthesis method	Filler content	Electrical conductivity (S/m)	Reference
PMMA/Graphene	Intercalation polymerization	4 wt %	17.55	[119]
		10 wt %	1719	
PMMA/rGO	In situ polymerization	3 wt %	1.5	[125]
PMMA/rGO	Aqueous mixing	2 wt %	3.7 × 10^−2^	[126]
PEO/Graphene	Aqueous mixing	2 wt %	6 × 10^−2^	[127]
PBT/rGO	Aqueous mixing	10 wt %	9 × 10^−2^	[128]
PET/Graphene	Melt mixing	7 wt %	~10^−4^	[129]
PI/rGO	In situ polymerization	30 wt %	11	[130]
Epoxy/Graphene foam	Prepreg-hot press	10 wt %	230	[131]

**Table 2:** EM wave absorption of different graphene/polymer composites.

Absorber	Synthesis method	Matrix	Absorber content	Thickness (mm)	RL min (dB)	RL < −10 dB bandwidth (GHz)	Reference
PPy/GO	Intercalation polymerization	Wax	30 wt %	2.96	−58.1	6.2	[120]
PANi/Graphene	Intercalation polymerization	Wax	10 wt %	3.5	−36.9	5.3	[53]
PPy/Graphene	Intercalation polymerization	Wax	10 wt %	2.7	−48.0	3.4	[117]
PANi/Graphene	In situ polymerization	Wax	25 wt %	3.04	−38.8	2.3	[132]
PEO/rGO	Aqueous mixing	PEO	2.6 vol %	1.8	−38.8	4.1	[133]
NBR/GO	Aqueous mixing	NBR	10 wt %	3	−57.0	4.5	[134]
PANi/Graphene foam	In situ polymerization	Graphene foam	-	2	−52.5	3.0	[135]
PANi/rGO	In situ polymerization	Wax	50 wt %	2	−41.4	4.2	[136]

**Table 3:** Improvement in the mechanical properties of composites synthesized by different methods.

Material	Synthesis method	Filler content	Mechanical properties relative to neat polymer (Δ*E*, Δ*E*’, Δσ_max_, Δ*K*_IC_) *	Reference
PMMA/Graphene	Intercalation polymerization	10 wt %	Δ*E*’ = 299% (at 45 °C)	[119]
TPU/GNR	Intercalation polymerization	0.5 wt %	Δ*E* = 70%, Δ*E*’ = 175% (at −25 °C), Δσ_max_ = 15%	[115]
Epoxy/GNR	Intercalation polymerization	0.15 wt %	Δ*E* = 11%, Δ*K*_IC_ = 43%	[106]
PVA/GO	Intercalation polymerization	0.04 wt %	Δ*E* = 43%, Δσ_max_ = 20%	[107]
PMMA/rGO	In situ polymerization	2 wt %	Δ*E* = 13%, Δσ_max_ = −41%	[137]
PMMA/Graphene	Twin screw extruding	20 wt %	Δ*E* = 7%, Δ*E*’ = 22% (at 100 °C), Δσ_max_ = 3%	[138]
Epoxy/rGO	Ball mill	2 wt %	Δ*E* = 5%, Δσ_max_ = 0%, Δ*K*_IC_ = 50%	[139]
Epoxy/Functioalized-GO	In situ polymerization	0.5 wt %	Δ*E* = 16%, Δσ_max_ = −75%, Δ*K*_IC_ = 33%	[140]
Thermoplastic polyurethane (TPU)/Graphene	Aqueous mixing	3 wt %	Δ*E* = 43%, Δσ_max_ = −22%	[141]

* Δ*E*: maximum Young’s modulus improvement; Δ*E*’: maximum storage modulus improvement; Δσ_max_: maximum tensile strength improvement; Δ*K*_IC_: maximum fracture toughness improvement.

**Table 4:** Thermal conductivity polymer/graphene composites synthesized by different methods.

Material	Synthesis method	Filler content	TC (W/(m·K))	TC enhancement compared to neat polymer	Reference
PA-6/Graphite nanoflakes	Intercalation polymerization	12 wt %	2.49	678%	[18]
PA-6/rGO	In situ polymerization	10 wt %	0.416	112%	[143]
PA-6/Graphene foam	In situ polymerization	2 wt %	0.847	300%	[144]
PA-6/Graphene-GO	In situ polymerization	10 wt %	2.14	569%	[142]
PA-6/Graphite	Twin screw extruding	30 wt %	1.37	350%	[145]
PS/Graphite nanoflakes	Melt mixing	~9.2 wt %	0.9	398%	[146]
PP/Graphite nanoflakes	Aqueous mixing	10 wt %	1.53	595%	[147]
PVA/Graphite nanoflakes	Aqueous mixing	10 wt %	1.43	580%	[147]
PBT/Graphite nanoflakes	In situ polymerization	20 wt %	1.98	1320%	[148]

**Table 5:** Other applications of graphene/polymer composites synthesized by intercalation polymerization.

Application	Material	Description	Reference
Sensing of serotonin	PLA/GO	Electrochemical detection with high concentration range (0.1–100.0 µM) and low detection limit (0.08 µM, where *s*/*n* = 3)	[149]
Sensing of methanol	PANi/GO	High sensitivity (Δ*R*/*R*_0_ = 20.9–37) for methanol vapor (100–500 ppm) *	[150]
Electrochemical supercapacitor	PANi/GO	High specific capacitance of 543.75 F/g and reversible electrochemical response up to 150th repeated cycles	[151]
Water vapor barrier	PVA/GO	Water vapor permeability declines about 5-fold to 0.66 × 10^−12^ g·cm·(cm^2^·s·Pa)^−1^ by adding 0.04 wt % GO	[107]
Nitrogen gas barrier	TPU/GNR	Nitrogen gas effective diffusivity decreased by 3 orders of magnitude with only 0.5 wt % GNRs.	[115]

* Δ*R*/*R*_0_ = (*R* − *R*_0_)/*R*_0_, where, *R*_0_ and *R* are the initial resistance of sensor in the air and in target gas, respectively.

## Abbreviations

CVD	Chemical vapor deposition
NG	Natural graphite
GO	Graphene oxide
GtO	Graphite oxide
GIC	Graphite intercalated compounds
Pyr_14_TFSI	*N*-butyl-*N*-methylpyrrolidinium bis-(trifluoromethanesulfonyl)imide
PC	Propylene carbonate
DMSO	Dimethyl sulfoxide
DMF	Dimethylformamide
Pyr_14_^+^	*N*-butyl-*N*-methylpyrrolidinium cation
LiTFSI	Lithium bis(trifluoromethanesulfonyl)imide
TFSI^−^	Bis(trifluoromethanesulfonyl)imide anion
TBA^+^	Tetrabutylammonium cations
1,2-DAP	1,2-diaminopropane
XRD	X-ray diffraction
ANi^+^	Aniline cation
CL^+^	Caprolactam onium ion
EOG	Edge-selectively oxidized graphite
C_14_N^+^	Tetradecyl-ammonium cation
TAA^+^	Tetraalkylammonium ions
PVA	Poly(vinyl alcohol)
PDDA	Poly(diallyldimethylammonium chloride)
PVAc	Poly(vinyl acetate)
EG	Expanded graphite
ANi	Aniline
KC_24_	Stage 2 potassium intercalated graphite
KC_8_	Stage I potassium intercalated graphite
GNRs	Graphene nanoribbons
MWCNTs	Multiwalled carbon nanotubes
PANi	Polyaniline
PPy	Polypyrrole
PA-6	Polyamide-6
CEG	Chemical expanded graphite
PMMA	Polymethyl methacrylate
SEM	Scanning electron microscope
TEM	Transmission electron microscope
AFM	Atomic force microscope
FTIR	Fourier transform infrared
XPS	X-ray photoelectron spectroscopy
DSC	Differential scanning calorimetry
POM	Polarized optical microscopy
rGO	Reduced graphene oxide
PEO	Polyethylene oxide
PBT	Poly(butylene terephthalate)
PET	Poly(ethylene terephthalate)
PI	Polyimide
EM	Electromagnetic waves
RL	Refection loss
NBR	Nitrile butadiene rubber
TPU	Thermoplastic polyurethane
TC	Thermal conductivity
PS	Polystyrene
PP	Polypropylene
PLA	Poly(lactic acid)
